# Clinical significance of cripto-1 expression in lung adenocarcinoma

**DOI:** 10.18632/oncotarget.15761

**Published:** 2017-02-27

**Authors:** Hua Zhang, Bin Zhang, Liuwei Gao, Lianmin Zhang, Kaikai Zhu, Runfen Cheng, Changli Wang

**Affiliations:** ^1^ Department of Lung Cancer, Tianjin Medical University Cancer Institute and Hospital, Tianjin, China; ^2^ National Clinical Research Center for Cancer, Tianjin, China; ^3^ Key Laboratory of Cancer Prevention and Therapy, Tianjin, China; ^4^ Tianjin Lung Cancer Center, Tianjin, China; ^5^ Department of Pathology, Tianjin Medical University Cancer Institute and Hospital, Tianjin, China

**Keywords:** Cripto-1, immunohistochemistry, lung adenocarcinoma, epidermal growth factor receptor, prognosis

## Abstract

Cripto-1 can promote tumourigenesis and may be a potential prognostic biomarker in several malignancies, yet little is known about this protein in lung adenocarcinoma (LAC). The aim of this study was to evaluate the prognostic value of cripto-1 expression in a cohort of patients with LAC. Tumours from 290 patients with pathologically confirmed LAC were used for an immunohistochemical analysis of cripto-1 expression. The correlation between cripto-1 expression and the clinicopathological parameters of patients, EGFR-TKI sensitivity was analysed. Significant associations between cripto-1 expression and pT status, pN status, pTNM status, E-cadherin expression and EGFR-TKI sensitivity were identified. Compared with patients with low cripto-1 expression, patients with high cripto-1 expression exhibited significantly poorer progression-free survival (PFS) and overall survival (OS). Moreover, multivariate analyses showed that high cripto-1 expression was an independent predictor of worse survival of patients with LAC. The combination of cripto-1 expression and serum CEA level was correlated with both PFS and OS. In conclusion, cripto-1 may be a potential prognostic biomarker of survival in patients with LAC.

## INTRODUCTION

Lung cancer is the leading cause of cancer-related mortality worldwide. In China, lung cancer has become the second leading cause of death after liver cancer [1]. Non-small cell lung cancer (NSCLC) accounts for 80% of all cancers and includes adenocarcinoma, squamous cell carcinoma, and large cell carcinoma. Previous studies have reported that lung adenocarcinoma (LAC) grows and spreads faster than lung squamous cell carcinoma and constitutes almost half of all lung cancers [2]. Despite advances in multi-modal treatment strategies, the 5-year survival rate of patients with NSCLC remains lower than 15% [3]. Recently, targeted therapies have revolutionized the management of lung cancer patients, but these therapies are restricted to select cases due to infrequently characterized driver mutations [4-5]. Hence, it is essential to identify novel prognostic markers and therapeutic targets.

Cripto-1, which is a member of the glycosylphosphatidylinositol (GPI)-anchored signalling protein family [6, 7], acts as a coreceptor for the transforming growth factor-β (TGF-β) subfamily of ligands Nodal and growth differentiation factor-1 and -3 (GDF1 and GDF3). Cripto-1 is critically important in early embryogenesis, stem cell maintenance and malignant progression [8]. In addition, cripto-1 has been reported to play a role in epithelial-mesenchymal transition (EMT) and as a stem cell regulator [8, 9]. Cripto-1 overexpression is also correlated with poor prognosis in gastric cancer, bladder cancer, and hepatocellular carcinoma [10-12]. However, little is known about the prognostic value of cripto-1 in NSCLC [13]. Shan *et al.* investigated the correlation between cripto-1 expression and clinicopathological parameters in patients with LAC [14]. Until now, the prognostic value of cripto-1 in patients with LAC has never been studied. In the present study, we analysed the expression of cripto-1 in LAC tissues by immunohistochemistry (IHC). The correlation between cripto-1 expression and clinicopathological parameters and the survival of patients with LAC was analysed. A recent study demonstrated that cripto-1 expression in EGFR-mutant NSCLC elicits intrinsic EGFR-TKI resistance [15]. Therefore, we also investigated the association between cripto-1 expression and EGFR-TKI sensitivity.

## RESULTS

### The characteristics of LAC patients

In total, 132 male and 158 female patients with a median age of 58 years (range, 33 years to 77 years) were included in this study. Of these patients, 171 were non-smokers, and 119 were current or former smokers. The distribution of patients with respect to pathologic tumour stage according to the seventh edition of the tumour-node-metastasis (TNM) classification revealed 114 stage I, 30 stage II, 104 stage III, and 42 stage IV. EGFR mutation detection was performed in all patients in the entire cohort, and 131 patients were positive for EGFR mutations. Among the 131 EGFR mutations, 65 were deletion in exon 19 and 66 were the L858R mutation in exon 21. Anti-EGFR drugs (erlotinib or gefitinib) were used in 93 cases. The follow-up period ranged from 5 months to 83 months (median, 44.0 months; mean, 44.3 months). The clinicopathological characteristics of the patients are listed in Table 1.

**Table 1:** Baseline characteristics of all LAC patients.

Characteristics	Number of patients	Percentage (%)
Age(years)		
Median(range)	58(33-77)	
≥58	147	50.7%
<58	143	49.3%
Sex		
female	158	54.5%
male	132	45.5%
Smoking status		
yes	119	41.0%
no	171	59.0%
pT status		
T1	158	54.5%
T2	92	31.7%
T3	5	1.7%
T4	3	1.1%
Unknown	32	11.0%
pN status		
N0	133	45.9%
N1	21	7.2%
N2	103	35.5%
N3	16	5.5%
Unknown	17	5.9%
pM status		
M0	248	85.5%
M1	42	14.5%
pTNM status		
I	114	39.3%
II	30	10.3%
III	104	35.9%
IV	42	14.5%
EGFR mutation status		
positive	131	45.2%
negative	159	54.8%
Anti-EGFR drug treatment		
yes	93	32.1%
no	197	67.9%

### Expression of cripto-1 in LAC and its relationship with clinicopathological parameters

**Figure 1:**
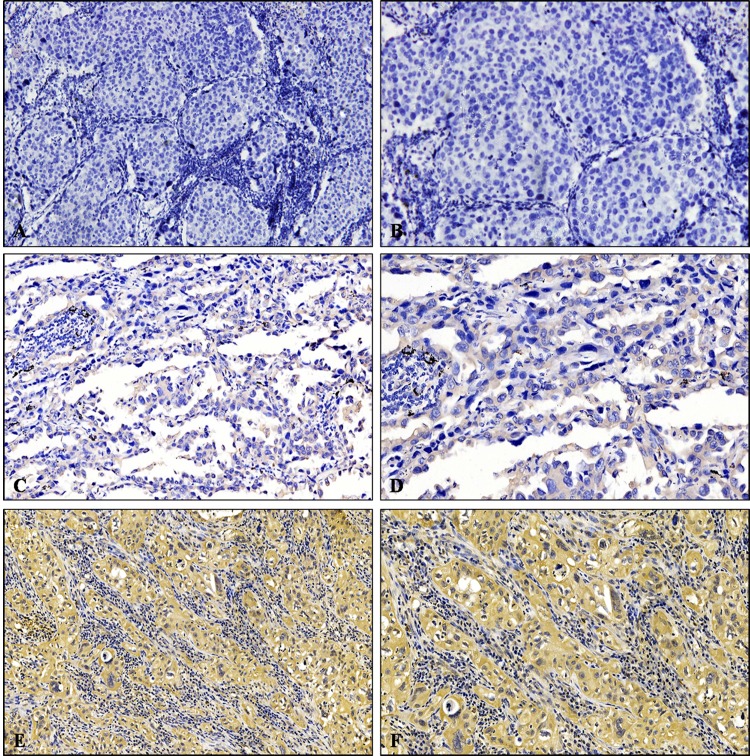
Immunohistochemical analysis of cripto-1 expression in LAC patients **A.** and **B.** Negative expression of cripto-1. **C.** and **D.** Low expression level of cripto-1. **E.** and **F.** High expression level of cripto-1. **A.**, **C.**, and **E.** Original magnification×100; **B.**, **D.**, and **F.** Original magnification×200.

Specific cripto-1 staining was localized predominantly in the cytoplasm of the tumour cells (Figure 1). In addition, positive staining in the nuclei of tumour cells was also detected. According to the cripto-1 immunoreactivity score, high cripto-1 expression was found in 157 of the 290 LAC patients, while 133 (45.9%) patients were classified as the low cripto-1 group. The relationship between cripto-1 expression and the clinicopathological parameters is shown in Table 2. Our results demonstrated that cripto-1 expression in LAC was associated with pT status (*P* = 0.047), pN status (*P* < 0.001), pTNM status (*P* = 0.001), and E-cadherin expression (*P* = 0.001). In contrast, cripto-1 expression displayed no association with age, sex, smoking status, tumour location, lesion type, pM status, EGFR mutation status or serum CEA level.

**Table 2:** Correlation of cripto-1 protein expression with clinicopathological parameters.

Characteristics	Cripto-1(low)	Cripto-1(high)	*P* value
Age (years)			
≥58	69	78	
<58	64	79	0.709
Sex			
female	78	80	
male	55	77	0.190
Smoking status			
yes	51	68	
no	82	89	0.392
Tumour location			
left	53	72	
right	80	85	0.303
Lesion type			
central	57	75	
peripheral	76	82	0.402
pT status			
T1	80	78	
T2+T3+T4	38	62	0.047
pN status			
N0	77	56	
N1+N2+N3	48	92	<0.001
pM status			
M0	111	137	
M1	22	20	0.359
pTNM status			
I+II	80	64	
III+IV	53	93	0.001
EGFR mutation status			
yes	65	66	
no	68	91	0.244
Serum CEA			
normal (≤5 ng/mL)	79	80	
elevated (>5ng/mL)	54	77	0.150
E-cadherin			
negative	63	105	
positive	70	52	0.001

### High cripto-1 expression is inversely associated with EGFR-TKI sensitivity in LAC

In order to investigate the clinical significance of cripto-1 in EGFR-TKI sensitivity, we analysed a cohort of 93 LAC patients who had received EGFR-TKI treatment (erlotinib or gefitinib), either as front line or salvage treatment. According to the definition of EGFR-TKI sensitivity, 79 patients were sensitive to EGFR-TKI, and 14 patients were intrinsically resistant to EGFR-TKI. The correlation of cripto-1 expression with EGFR-TKI sensitivity is shown in Table 3. Thirteen of 14 intrinsically resistant tumours showed high cripto-1 expression. In the contrast, 46 of 79 sensitive tumours exhibited low cripto-1 expression. Our results demonstrated that compared with sensitive patients, a higher proportion of patients who were intrinsically resistant to EGFR-TKI treatment exhibited high cripto-1 expression.

**Table 3:** High cripto-1 expression is inversely associated with EGFR-TKI sensitivity in LAC.

	Cripto-1 (low)	Cripto-1 (high)	*P* value
EGFR-TKI			
responder	46	33	
non-responder	1	13	<0.001

### The association between cripto-1 expression and survival in LAC patients

**Figure 2:**
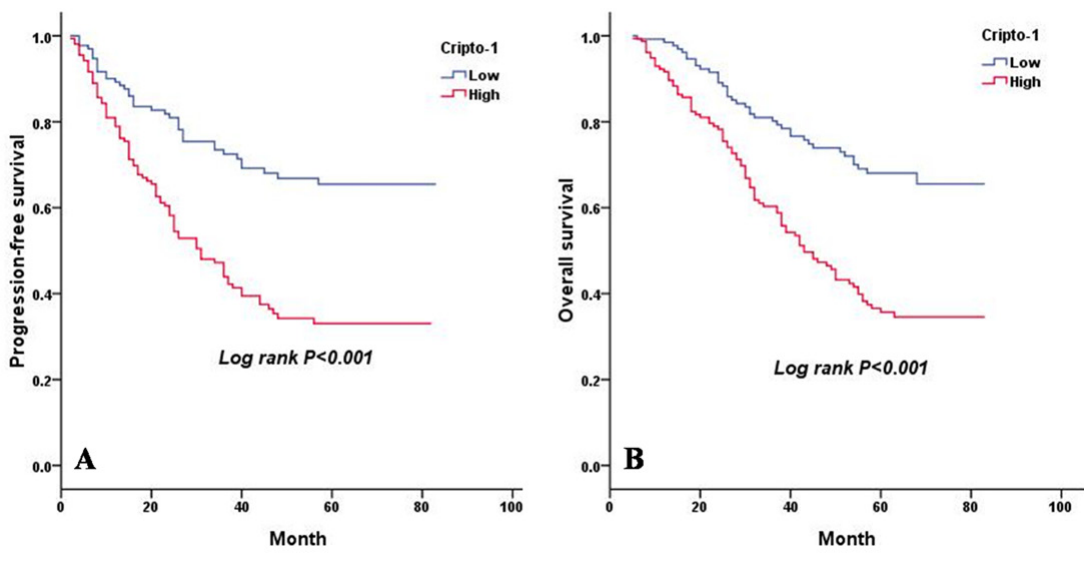
PFS and OS are shown for 290 patients with LAC **A.** PFS curves for 290 patients with LAC according to cripto-1 expression. **B.** OS curves for 290 patients with LAC according to cripto-1 expression.

**Figure 3:**
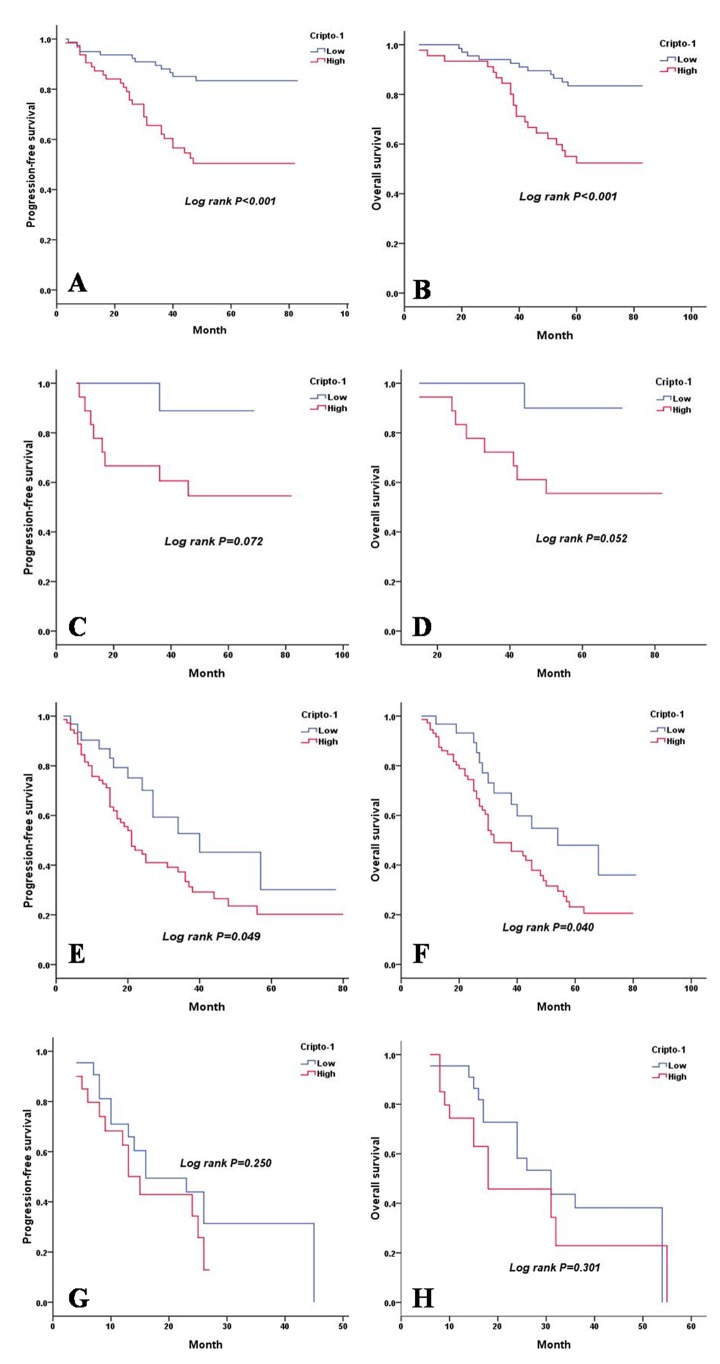
PFS and OS are shown for stage I, II, III and IV LAC patients **A.** PFS curves of stage I LAC patients according to cripto-1 expression. **B.** OS curves of stage I LAC patients according to cripto-1 expression. **C.** PFS curves of stage II LAC patients according to cripto-1 expression. **D.** OS curves of stage II LAC patients according to cripto-1 expression. **E.** PFS curves of stage III LAC patients according to cripto-1 expression. **F.** OS curves of stage III LAC patients according to cripto-1 expression. **G.** PFS curves of stage IV LAC patients according to cripto-1 expression. **H.** OS curves of stage IV LAC patients according to cripto-1 expression.

As shown in Table 4, a univariate analysis revealed that lesion type (*P* = 0.014 and *P* = 0.015), pTNM status (*P* < 0.001 and *P* < 0.001), serum CEA level (*P* < 0.001 and *P* < 0.001), E-cadherin expression (*P* = 0.026 and *P* = 0.028), and cripto-1 expression (*P* < 0.001 and *P* < 0.001) were each significantly associated with PFS and OS. The 5-year PFS rate and the median survival time of patients in the high cripto-1 group were significantly poorer compared with patients in the low cripto-1 group (33.0% *vs* 65.5% [24.0 *vs* 39.0 months], *P* < 0.001; Figure 2A). The 5-year OS rate was 35.7% for the high cripto-1 expression group and 68.0% for the low cripto-1 expression group, and the median survival time was 37.0 and 58.0 months, respectively, for the high and low expression groups (*P* < 0.001; Figure 2B). When the analysis was stratified by pathological stage (I, II, III, IV), we found that PFS and OS were better in the low cripto-1 expression group compared with the high cripto-1 expression group for the pathological stage I, II, and III subgroups (stage I: *P* < 0.001 for PFS, *P* < 0.001 for OS, Figure 3A, 3B; stage II: *P* = 0.072 for PFS, *P* = 0.052 for OS, Figure 3C, 3D, stage III: *P* = 0.049 for PFS, *P* = 0.040 for OS, Figure 3E, 3F). However, in stage IV patients, no significant correlation was found between cripto-1 expression and survival (*P* = 0.250 for PFS and *P* = 0.301 for OS, Figure 3G, 3H).

**Table 4:** Univariate analysis of PFS and OS for all LAC patients.

	PFS			OS		
	95%CI	HR	*P* value	95%CI	HR	*P* value
Age(≥58,<58)	0.752-1.502	1.063	0.730	0.804-1.605	1.136	0.470
Sex(female, male)	0.517-1.031	0.730	0.074	0.510-1.018	0.726	0.054
Smoking status(yes, no)	0.568-1.150	0.808	0.237	0.612-1.238	0.870	0.440
Tumour location(left, right)	0.584-1.169	0.827	0.281	0.596-1.193	0.843	0.335
Lesion type(central, peripheral)	1.091-2.182	1.543	0.014	1.086-2.171	1.536	0.015
pTNM status(I+II,III+IV)	2.822-6.063	4.136	<0.001	2.950-6.317	4.137	<0.001
EGFR mutation status(yes, no)	0.813-1.650	1.158	0.416	0.785-1.590	1.117	0.539
Serum CEA(≤5 ng/mL, >5 ng/mL)	1.383-2.776	1.959	<0.001	1.315-2.639	1.863	<0.001
E-cadherin(negative, positive)	0.457-0.952	0.660	0.026	0.460-0.958	0.664	0.028
Cripto-1(low, high)	1.734-3.688	2.529	<0.001	1.742-3.700	2.539	<0.001

PFS progression-free survival, OS overall survival, CI confidential interval, HR hazard ratio.

**Figure 4:**
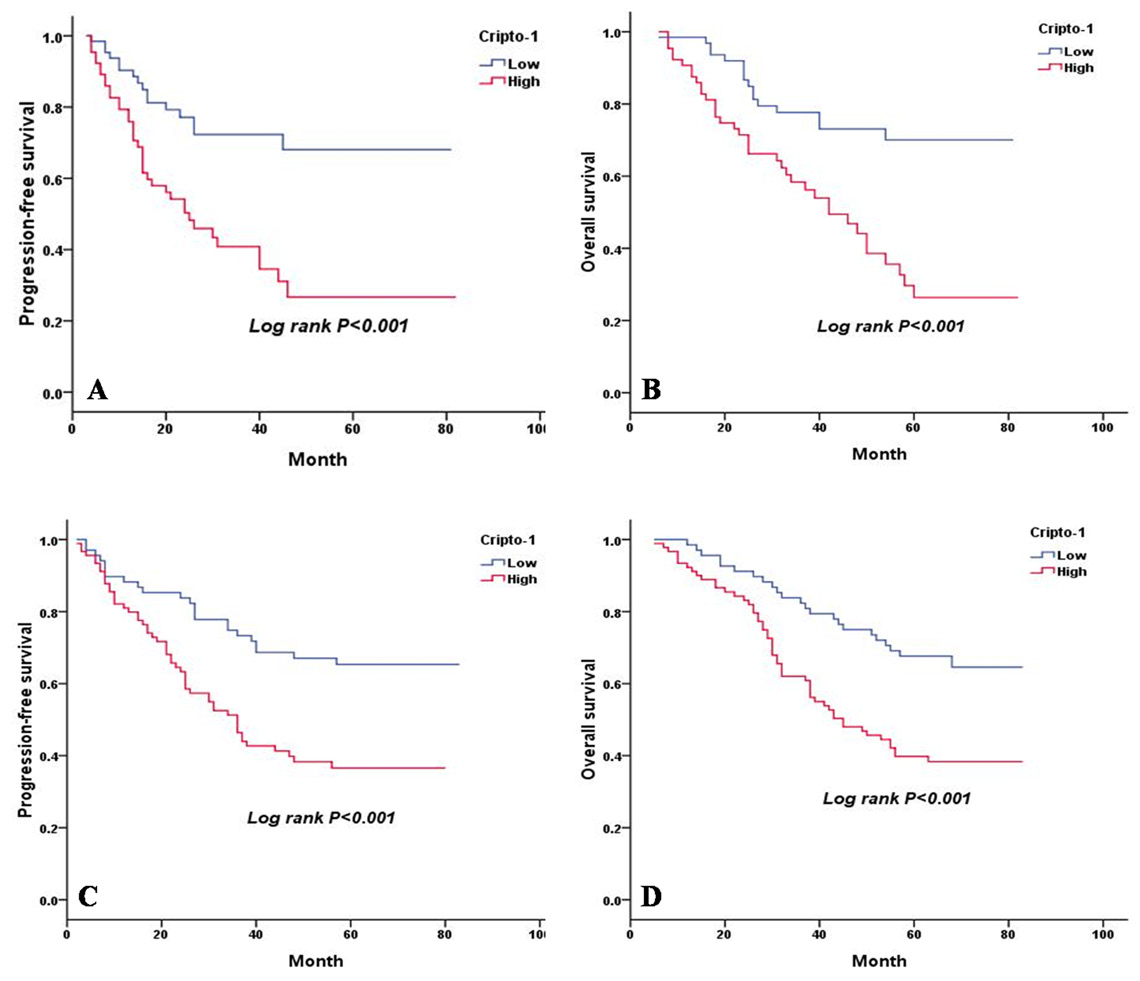
PFS and OS are shown for LAC patients with and without EGFR mutation **A.** PFS curves for LAC patients with mutated EGFR according to cripto-1 expression. **B.** OS curves for LAC patients with mutated EGFR according to cripto-1 expression. **C.** PFS curves for LAC patients with wild-type EGFR according to cripto-1 expression. **D.** OS curves for LAC patients with wild-type EGFR according to cripto-1 expression.

Based on EGFR mutation status, we found that high cripto-1 expression was significantly associated with shorter PFS and OS in the patients who had mutated EGFR (PFS: *P* < 0.001, Figure 4A, OS: *P* < 0.001, Figure 4B). For patients who had wild-type EGFR, we also observed a similar correlation between low cripto-1 expression and better prognosis (PFS: *P* < 0.001, Figure 4C, OS: *P* < 0.001, Figure 4D).

In order to assess independent prognostic indicators, those that were significant (*P* < 0.05) based on a univariate analysis were enrolled in a multivariate analysis (Table 5). Our results revealed that cripto-1 overexpression was a poor independent predictor of PFS (hazard ratio [HR] = 1.925, 95%CI: 1.295 to 2.862, *P* = 0.001) and OS (HR = 1.912, 95%CI: 1.290 to 2.833, *P* = 0.001) in patients with LAC.

**Table 5:** Multivariate analysis of PFS and OS for all LAC patients.

	PFS			OS		
	95%CI	HR	*P* value	95%CI	HR	*P* value
Lesion type(central, peripheral)	0.884-1.790	1.258	0.203	0.886-1.788	1.259	0.199
pTNM status(I+II,III+IV)	2.121-4.838	3.203	<0.001	2.260-5.516	3.414	<0.001
Serum CEA(≤5 ng/mL, >5 ng/mL)	0.866-1.818	1.254	0.231	0.814-1.714	1.181	0.381
E-cadherin(negative, positive)	0.606-1.304	0.889	0.548	0.591-1.264	0.864	0.452
Cripto-1(low, high)	1.295-2.862	1.925	0.001	1.290-2.833	1.912	0.001

PFS progression-free survival, OS overall survival, CI confidential interval, HR hazard ratio.

### The effect of combined cripto-1 expression and serum CEA level on the prognosis of LAC patients

**Figure 5:**
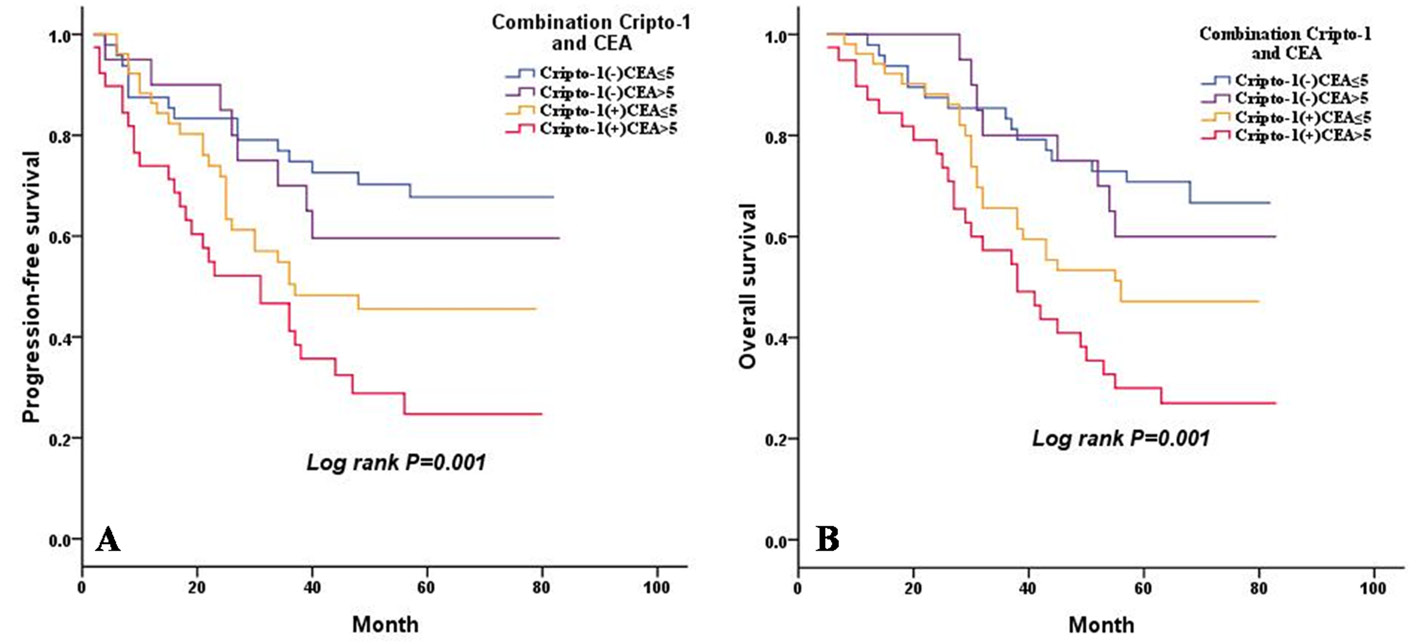
Combined influence of cripto-1 and CEA level on risk of LAC progression and death **A.** PFS curves for LAC patients according to the combination of cripto-1 expression and CEA level. **B.** OS curves for LAC patients according to the combination of cripto-1 expression and CEA level.

It has been shown that serum CEA level is a prognostic factor in patients with LAC [16]. In our study, a univariate analysis showed that a pretreatment serum CEA level above 5 ng/mL was significantly associated with poorer PFS (*P* < 0.001) and OS (*P* < 0.001) (Table 4). Therefore, we evaluated the combination effect of cripto-1 expression and serum CEA level on the progression and survival of patients with LAC. Based on cripto-1 expression and serum CEA level, all LAC patients were categorized into four groups with different progression risks and prognosis: group I, cripto-1 (-) and a CEA level ≤5 ng/mL, good prognosis and low-risk of progression; group II, cripto-1 (-) and a CEA level > 5 ng/mL and group III, cripto-1 (+) and a CEA level ≤5 ng/mL, intermediate prognosis and intermediate-risk of progression; group IV, cripto-1 (+) and a CEA level > 5 ng/mL, poor prognosis and high-risk of progression (Figure 5).

## DISCUSSION

In this study, we investigated the expression of cripto-1 and its value in the prediction of clinical outcomes in patients with LAC. The correlation between cripto-1 expression and the clinicopathological parameters of patients, EGFR-TKI sensitivity was also analysed. To the best of our knowledge, this is the first study to evaluate the value of cripto-1 as a prognostic indicator in patients with LAC.

Cripto-1, which is a member of the EGF-CFC family, regulates essential steps in early embryogenesis and plays an important role not only in early embryogenesis but also in stem cell maintenance and malignant progression [8]. Expression of cripto-1 protein has been investigated in human tumours including gastric cancer [10], bladder cancer [11], and hepatocellular carcinoma [12], NSCLC [13, 14]. Xu *et al.* reported that cripto-1 expression in NSCLC was 54.7% [13]. Shan *et al.* performed an immunohistochemical study of cripto-1 in 188 patients with LAC and found that the positive expression rate of cripto-1 was 72.3% [14]. However, the rate of cripto-1 expression was 54.1% in our study. The variability in these data may be partially explained by two reasons: first, the patients who were enrolled in the different studies may have had tumours that exhibited a heterogeneous cell population; second, different studies may have used different antibodies, immunohistochemical staining techniques and scoring systems.

Small molecule inhibitors of receptor tyrosine kinases are currently an important treatment for NSCLC, especially for patients that harbour an activating mutation in EGFR. Patients with EGFR mutations are highly sensitive to EGFR-TKIs [17]. EGFR-sensitizing mutations have been used to select patients with advanced NSCLC for EGFR-TKI treatment. Although clinical response to EGFR-TKIs is impressive, approximately 10% of NSCLC patients harbouring EGFR-sensitizing mutations exhibit primary resistance [18]. The mechanisms of primary resistance to EGFR-TKIs in the presence of sensitizing mutations are relatively known. Park KS *et al.* analysed cripto-1 expression by IHC in 85 EGFR mutant NSCLC specimens and found that cripto-1 expression was significantly higher in patients with primary resistance compared with patients who were sensitive [15]. In their study, cripto-1 expression was grouped according to staining intensity, and all (15/15) primary resistant tumours exhibited some degree of cripto-1 expression. In our study, the degree of immunoreactivity for cripto-1 was evaluated semiquantitatively on the basis of staining intensity and the proportion of positive cells. Consistent with previous results, our study also demonstrated that high cripto-1 expression is associated with EGFR-TKI primary resistance in LAC patients who harbour activating EGFR mutations. However, the patient numbers in the two studies, especially the number of cases with primary resistance to EGFR-TKIs, were relatively small, and therefore, additional large-cohort studies are needed to confirm these findings. Aside from that, further studies are needed to elucidate the mechanism by which cripto-1 participates in the primary resistance to EGFR-TKIs and to address whether cripto-1 may be used as a target for therapeutic approaches.

E-cadherin, which is a calcium-dependent cell-cell adhesion molecule that binds to β-catenin to form a complex that promotes adhesion, is expressed on the membranes of epithelial cells. E-cadherin expression is a hallmark of EMT [19]. One study showed that cripto-1 overexpression can induce EMT in mammary epithelial cell lines [20]. Not only can cripto-1 induce EMT properties *in vitro*, but Mouse Mammary Tumour Virus (MMTV)-Cripto transgenic mice form tumours that display EMT features including the downregulation of E-cadherin and the upregulation of vimentin and N-cadherin [21]. Consistent with previous studies, our study demonstrated a negative correlation between cripto-1 and E-cadherin expression. Shibamoto S *et al.* reported that tyrosine phosphorylation of β-catenin can inhibit its association with E-cadherin and facilitate cell scattering and migration [22]. Compared with controls, cell lines that overexpress cripto-1 exhibit an increased basal level of tyrosine phosphorylation of β-catenin, which leads to the decreased association of β-catenin and E-cadherin [23]. This may show a mechanistic relationship between cripto-1 expression and the loss of E-cadherin expression.

A Kaplan-Meier survival analysis showed that PFS and OS of patients with a high cripto-1 expression were significantly poorer than the PFS and OS of patients with low cripto-1 expression. According to the results of the multivariate analysis, we showed that cripto-1 overexpression was an independent predictor of poor PFS as well as OS in patients with LAC, which is comparable to the results observed in NSCLC [13]. When we compared the effect of cripto-1 expression in patients with different disease stages, we found a significant association between cripto-1 expression and both PFS and OS in patients with stages I and III disease. In addition, a borderline significance was demonstrated in stage II patients. However, in stage IV patients, no significant correlation was found between cripto-1 expression and survival. First, a reason for these results may be the small number of patients with advanced disease in our study. Second, compared with advanced LAC, cripto-1 may have more predictive value in non-advanced LAC. Therefore, the prognostic value of cripto-1 in advanced LAC requires further study. Next, the stratification of the analyses according to EGFR status revealed that the significant effect was observed in patients with EGFR mutations and in those with wild-type EGFR. It has been shown that LAC patients with CEA levels higher than 5 ng/mL have a poorer survival and an increased risk of locoregional recurrence [16]. However, for patients with normal CEA levels, it is difficult to predict prognosis and progression. In our study, 50.3% (80/159) of patients with normal CEA levels had tumours with high cripto-1 expression. Thus, we divided the LAC patients into four groups according to cripto-1 expression and CEA level and found that the combination of cripto-1 expression and CEA level could be used to predict the progression of tumours and the survival of patients. Cripto-1 is a secreted protein, and recently, Xu CH *et al.* reported that the serum level of cripto-1 is a useful diagnostic biomarker and an independent prognostic biomarker of survival in patients with NSCLC [24]. Patients with lung cancer usually undergo full blood testing, and thus, it is easy to obtain the serum cripto-1 and CEA level. Moreover, blood tests are non-invasive and hematologic markers are much cheaper and faster to detect.

Our study does have several potential limitations. First, our study is a retrospective study, and all data are from a single institution. Therefore, the selection bias cannot be fully excluded. Second, the number of patients with advanced LAC was relatively small, and thus, the prognostic value of cripto-1 in patients with advanced disease requires a larger study. In addition, all patients enrolled in the present study are Chinese, and we do not know if these findings are generalizable worldwide. Therefore, further studies are required to confirm the results of this study.

In summary, based on the results of our study, cripto-1 expression is able to predict the prognosis of patients with LAC. Additionally, high cripto-1 expression is significantly correlated with EGFR-TKI primary resistance. However, the mechanism of primary resistance associated with cripto-1 and the prognostic value of serum cripto-1 expression require further investigation.

## MATERIALS AND METHODS

### Patients

Between May 2009 and September 2013, a total of 325 consecutive LAC patients received treatment at the Department of Lung cancer, Tianjin Medical University Cancer Institute and Hospital. Patients were excluded from the study if they met the following criteria: (1) receipt of preoperative chemotherapy or radiotherapy (*n* = 8); (2) survival of less than 3 months after surgery (*n* = 6); (3) history of lung cancer or a second primary cancer diagnosed within 5 years of the lung cancer index (*n* = 6). Patients who could not provide enough tumour tissues for immunohistochemistry (*n* = 15) were also excluded from the analyses. Ultimately, 290 patients with LAC, all of whom were Chinese, were enrolled in our study. Moreover, 240 of 290 patients underwent complete resection and systematic node dissection of the hilar and mediastinal lymph nodes. Fifty specimens were obtained from patients with stage IIIB to IV disease. These specimens were obtained through supraclavicular lymph node biopsy, pleural biopsy, fibre bronchoscope biopsy, computed tomography or with ultrasonic-guided percutaneous transthoracic biopsy. The pathological diagnosis of LAC was determined according to the revised World Health Organization Classification of Lung Tumours [25]. The stage of each patient was determined according to the seventh edition of the tumour-node-metastasis (TNM) classification system [26]. All patients signed an informed consent, and this study was approved by the ethics committee of Tianjin Medical University Cancer Institute and Hospital.

### Immunohistochemistry (IHC)

Formalin-fixed paraffin-embedded (FFPE), 4-µm-thick tissue sections were used for the immunohistochemical analysis. IHC was performed as described previously [27]. Paraffin sections were first deparaffinised in xylene and rehydrated in serially decreasing concentrations of ethanol. After antigen retrieval in 0.01 M sodium citrate buffer (pH = 6.0) in a microwave, endogenous peroxidase activity was blocked by incubation of the slides in 3% H_2_O_2_ for 10 min. Then, tissue sections were incubated with rabbit anti-cripto1 (Epitomics and Rockland, 1:1000) and rabbit anti-E-cadherin (Invitrogen, 1:200) antibodies overnight at 4°C. This was followed by incubation of the slides with the corresponding secondary antibodies for 30 min at 37°C, after which point the sections were washed with PBS and incubated for 1 min with 3,3’-diaminobenzidine (DAB). All sections were counterstained in haematoxylin and were dehydrated, cleared and permanently mounted with resinous mounting medium. All the procedures were performed at room temperature. Additionally, tissue that stained positive for cripto-1 was used as positive control. An unrelated rabbit IgG mAb was used as the negative control at the same dilutions as the corresponding primary antibody.

### Quantification of immunohistochemical staining

Two investigators evaluated the samples independently and were unaware of the clinical data of all patients. The scoring of cripto-1 expression was conducted according to the intensity of staining and the proportions of positive tumour cells: (a) staining intensity score (score of 0, negative staining; score of 1, weak staining, light yellow; score of 2, intermediate staining, yellow brown; and score of 3, strong staining), (b) the percentage of positive tumour cells (score of 0, < 5%; score of 1, 5%-20%; score of 2, 21%-50%; score of 3, 51%-75%; score of 4, > 75%). Cripto-1 expression was calculated by a+b, which means that the score ranged from 0 to 7. The samples were then categorized into two groups according to those scores, as follows: a score of less than or equal to 3 indicated low expression, and a score greater than 3 indicated high expression. The scoring criteria for E-cadherin in NSCLC have been described previously [28]. When ≥50% of the carcinoma cells in a given specimen were positively stained for E-cadherin, the sample was classified as E-cadherin-positive. Conversely, when < 50% of the cells were stained, the specimen was classified as E-cadherin-negative. If a discrepancy occurred with some samples, then the slides were reviewed again, and a consensus was reached.

### Definition of the sensitivity to EGFR-TKIs

Efficacy was evaluated after the first month of treatment, and subsequent evaluations were performed every 2 months. Routine follow-up assessments included physical examinations, chest CT, abdominal and cervical ultrasound, brain MRI, ECT and laboratory tests. Efficacy was assessed according to the RECIST 1.1 guidelines [29]. Patients with a complete response (CR), partial remission (PR), or stable disease (SD) more than 4-month duration were defined as sensitive cases. Patients with progressive disease (PD) or SD of 4-month duration or less were defined as intrinsically resistant cases [15].

### Statistical analysis

The associations between cripto-1 expression and clinicopathological parameters and EGFR-TKI sensitivity were evaluated with the chi-square test. OS was defined as the time between the treatment and the date of death from any cause or the last follow-up. PFS was defined as the time from treatment until tumour progression or the last follow-up. The Kaplan-Meier method and the log-rank test were used to calculate the OS and PFS. Univariate and multivariate analyses were based on the Cox proportional hazards regression model for independent prognostic value. All statistical analyses were performed using SPSS software (version 18.0; SPSS, Inc., Chicago, IL, USA). All statistical tests were two-sided, and statistical significance was accepted at the *P* < 0.05 level.
